# Why study design matters: evaluating bias and generalizability in contrast-enhanced mammography research

**DOI:** 10.1186/s13244-025-02139-7

**Published:** 2025-11-19

**Authors:** Marc B. I. Lobbes, Patricia J. Nelemans

**Affiliations:** 1 Department of Medical Imaging, Zuyderland Medical Center, Sittard-Geleen, The Netherlands; 2 Department of Epidemiology, Maastricht University, Maastricht, The Netherlands

Dear Editor,

We read with great interest the study by Pediconi et al, which evaluated the diagnostic performance of contrast-enhanced mammography (CEM) using a high-concentration iodinated contrast medium (HCCM) [1]. The study aimed to determine whether a reduced iodine volume—achieved through higher contrast concentration and lower injection volume—could provide a more sustainable alternative to conventional CEM protocols employing lower-concentration contrast media. The authors concluded that reducing the volume of iodinated contrast agent allows for a more sustainable approach to CEM without compromising diagnostic performance. This research question is of considerable importance, as reducing iodine load may benefit both patient safety and ecological impact. However, we believe that the study design imposes important limitations that affect the generalizability of the findings and preclude the definitive conclusions drawn by the authors regarding the applicability of an HCCM protocol.

In medical practice, diagnostic testing plays a vital role, and physicians rely on it to refine their clinical assessment. This reliance assumes that the tests yield dependable information about a patient’s health status. However, diagnostic tests often receive limited scrutiny, and studies evaluating them can show a plethora of biases [2]. Many tests are adopted into routine practice without undergoing thorough evaluation, and most published studies on diagnostic tests suffer from methodological issues that limit the validity and generalizability of the results. Consequently, tests with limited clinical value are frequently used, potentially exposing patients to potential harm—especially when tests produce inaccurate or non-reproducible results, or when diagnostic outcomes of specific populations are automatically implemented in other scenarios.

A methodological problem in this diagnostic study is the lack of a control group with a more standard contrast administration protocol (for example, 1.5 mL/kg with a dose of 300 mg/mL [3]). Consequently, a direct comparison to assess whether an HCCM protocol improves, maintains, or compromises diagnostic accuracy is impossible. Furthermore, the study included only women who, following initial digital mammography (DM) and ultrasound (US), were referred for CEM as a second-line imaging modality. Notably, 72.7% of patients were ultimately diagnosed with breast cancer—a prevalence far higher than would be expected in clinical populations. The predominance of ‘difficult’ cases, coupled with a high proportion of patients with dense breasts (85%), likely amplifies the perceived advantage of CEM over standard protocols and DM/US.

The study design introduces a type of selection bias known as work-up or verification bias. In a diagnostic study, verification of disease should occur in all patients independently of an initial positive or negative test result. This condition is not met in this study, where verification by the gold standard was only applied in a subsample of patients who were referred for US and CEM. Due to a referral that was based on the test result of DM, patients with a negative result on DM are underrepresented. Consequently, the selection leads to underestimation of false negative results and inflated estimates of CEM and US sensitivity, whereas specificity is often falsely reduced. Blackstone et al pointed to the fact that work-up bias is counterintuitive and therefore often poorly recognized [4]. Narrowing down a study to a defined subset of patients is necessary to evaluate the diagnostic performance of a test when used for its intended purpose. However, it makes extrapolation of results to a more general population treacherous. Comparison of biased estimates of accuracy with diagnostic performance of standard-concentration protocols as reported by studies in the literature is not valid because of differences in study populations.

Evidence that the use of the evaluated HCCM protocol does not compromise diagnostic performance must come from direct comparison with a control group in which a standard-concentration protocol is used. Nevertheless, Pediconi et al base their conclusion that HCCM is ‘sustainable without compromising performance’ on comparisons with existing literature. They mention the lack of direct comparison with a control group as a study limitation, but seem unaware that precisely this limitation is the reason that the study does not provide strong evidence for their conclusion.

In summary, although the study presents promising evidence that HCCM-CEM can achieve high diagnostic performance in a specific clinical setting, several methodological concerns—particularly the absence of a comparative control group and the selection of a disease-enriched population—necessitate caution in interpreting the results. These limitations constrain the extent to which conclusions about accuracy and sustainability can be generalized. At present, we believe the data do not justify replacing current CEM protocols with an HCCM-based approach in routine clinical or screening practice. Future prospective, controlled studies in more representative populations are warranted to confirm the findings and establish broader clinical applicability. Potential benefits of such an approach on sustainability can then also be studied separately.
